# Recombinant Laminin-511 Fragment (iMatrix-511) Coating Supports Maintenance of Human Nucleus Pulposus Progenitor Cells In Vitro

**DOI:** 10.3390/ijms242316713

**Published:** 2023-11-24

**Authors:** Hazuki Soma, Daisuke Sakai, Yoshihiko Nakamura, Shota Tamagawa, Takayuki Warita, Jordy Schol, Erika Matsushita, Mitsuru Naiki, Masato Sato, Masahiko Watanabe

**Affiliations:** 1 Department of Orthopedic Surgery, Tokai University School of Medicine, 143 Shimokasuya, Isehara 259-1193, Japan; soma.hazuki@tunzpharma.co.jp (H.S.); kahiko@is.icc.u-tokai.ac.jp (Y.N.); takayuki.warita@tunzpharma.co.jp (T.W.); schol.jordy@gmail.com (J.S.); me091130@tsc.u-tokai.ac.jp (E.M.); sato-m@is.icc.u-tokai.ac.jp (M.S.); masahiko@is.icc.u-tokai.ac.jp (M.W.); 2 TUNZ Pharma Corporation, Osaka 541-0046, Japan; m-naiki@nippon-zoki.co.jp; 3 Center for Musculoskeletal Innovative Research and Advancement (C-MiRA), Tokai University Graduate School, 143 Shimokasuya, Isehara 259-1193, Japan; 4 Department of Medicine for Orthopaedics and Motor Organ, Juntendo University Graduate School of Medicine, Tokyo 113-8421, Japan; s-tamagawa@juntendo.ac.jp

**Keywords:** intervertebral disc, nucleus pulposus, progenitor cell, Tie2, laminin-511, iMatrix-511, type 2 collagen, cell proliferation, regenerative potential

## Abstract

The angiopoietin-1 receptor (Tie2) marks specific nucleus pulposus (NP) progenitor cells, shows a rapid decline during aging and intervertebral disc degeneration, and has thus sparked interest in its utilization as a regenerative agent against disc degeneration. However, the challenge of maintaining and expanding these progenitor cells in vitro has been a significant hurdle. In this study, we investigated the potential of laminin-511 to sustain Tie2^+^ NP progenitor cells in vitro. We isolated cells from human NP tissue (*n* = 5) and cultured them for 6 days on either standard (Non-coat) or iMatrix-511 (laminin-511 product)-coated (Lami-coat) dishes. We assessed these cells for their proliferative capacity, activation of Erk1/2 and Akt pathways, as well as the expression of cell surface markers such as Tie2, GD2, and CD24. To gauge their regenerative potential, we examined their extracellular matrix (ECM) production capacity (intracellular type II collagen (Col2) and proteoglycans (PG)) and their ability to form spherical colonies within methylcellulose hydrogels. Lami-coat significantly enhanced cell proliferation rates and increased Tie2 expression, resulting in a 7.9-fold increase in Tie2-expressing cell yields. Moreover, the overall proportion of cells positive for Tie2 also increased 2.7-fold. Notably, the Col2 positivity rate was significantly higher on laminin-coated plates (Non-coat: 10.24% (±1.7%) versus Lami-coat: 26.2% (±7.5%), *p* = 0.010), and the ability to form spherical colonies also showed a significant improvement (Non-coat: 40.7 (±8.8)/1000 cells versus Lami-coat: 70.53 (±18.0)/1000 cells, *p* = 0.016). These findings demonstrate that Lami-coat enhances the potential of NP cells, as indicated by improved colony formation and proliferative characteristics. This highlights the potential of laminin-coating in maintaining the NP progenitor cell phenotype in culture, thereby supporting their translation into prospective clinical cell-transplantation products.

## 1. Introduction

Low back pain (LBP) is a prevalent issue that affects a substantial number of individuals worldwide [1,2] and demonstrates a strong association with intervertebral disc (IVD) degeneration. The IVD comprises three distinct tissue structures: the fibrocartilage ring, termed the annulus fibrosis, that encloses the central gelatinous nucleus pulposus (NP) tissue, and two cartilaginous endplates where the disc borders the vertebrae. Age-related disc degeneration is a progressive condition that may impair the stability of the spinal column, giving rise to a variety of spinal disorders [3,4]. This degenerative process of irreversible change is marked by a decline and deterioration in NP cells, resulting in changes in the extracellular matrix (ECM) composition, thereby compromising the disc’s biomechanical features [3,5]. The NP tissue plays a pivotal role in cushioning and distributing the stringent mechanical forces subjected to the IVD. Its ECM is rich in proteoglycans (PG) and type II collagen (Col2), which establish the disc’s remarkable biomechanical limits [6,7]. In addition, the cell-produced ECM provides chemical and structural support to endemic cells by regulating their motility, proliferation, differentiation, and viability, in part through integrin-mediated interactions [8,9,10]. As such, degeneration of IVD-ECM directly impacts cell behavior, likely further promoting a catabolic state. Recently, research efforts have been exploring regenerative medicinal strategies to halt or even reverse IVD degeneration, showing rapid progression in the last decade [11,12]. Specifically, cellular therapeutics have garnered much attention, with multiple clinical trials reporting pain and disability alleviation following intradiscal injection of de novo cell populations [13,14,15]. Different cell-type-based products are being explored, such as mesenchymal stromal cells or NP-derived cells, and are each speculated to have their advantages and hurdles for effective application [16,17].

In previous studies, we have reported on the presence of the angiopoietin-1 receptor tyrosine kinase (Tie2) on the surface of certain NP cells, which marks a specific progenitor cell population [18,19]. These Tie2^+^ NP cells exhibited remarkable cellular potency, characterized by enhanced proliferation rates [18,19,20], colony formation [21], ECM production (including high aggrecan and Col2 production) [22], and the maintenance of a high differentiation potential (e.g., neuronal, osteogenic, and adipogenic) [23,24,25]. For example, human Tie2^+^ NP cells, when seeded onto decellularized NP tissue fragments, were capable of repopulating and maintaining the NP tissue in a subcutaneous transplantation mouse model, highlighting their proliferative and differentiation potential [18]. Moreover, the induced differentiation of induced pluripotent stem (iPS) cells toward an active NP cell phenotype has shown an evident Tie2^+^ population in its initial stages of differentiation, underscoring its progenitor cell characteristics [26]. In human discs, the presence of Tie2 expression in NP cells showed a rapid age-dependent decline and was associated with the progression of disc degeneration [18,19]. Furthermore, our findings suggest that age negatively affects the functionality of Tie2^+^ NP cells [27], as evidenced by a decline in colony formation capabilities and proliferation rates [28]. Combining the proliferative characteristics and innate ability to survive within the relative harsh disc environments of these NP progenitor cells, as well as their evident loss during aging and degeneration progression, makes these Tie2^+^ NP cells an excellent candidate cell type for reintroduction into discs to potentially enhance and restore their structural integrity. Nevertheless, Tie2-expressing NP cells are notoriously difficult to maintain and expand in vitro, as NP cells tend to show a rapid decline in ECM production capacity, Tie2 expression, and overall proliferation rates with passaging [19,29]. As such, multiple strategies have been designed to optimize cell isolation and culture methods to increase yields and potency of these Tie2-expressing NP cells, including cell sorting methods [19], growth factor stimulation [22,25,30,31], cryostorage [21,32], and 3D culture environments [21,24]. Additional optimization is being explored to further support large-scale production of allogenic Tie2-expressing NP cells and to further increase the yield of NP progenitor cells from scarce young NP tissue explants without the need for complex and costly culture methods.

A pivotal factor known to maintain “stemness” of stem- and progenitor cells are the constituents laminin and integrin [9,33,34]. Within IVD tissues, a rich tapestry of laminin isoforms is expressed, exhibiting substantial variability in the distribution of binding proteins across different regions [35]. The intricate interplay between cells and basement membranes is widely acknowledged to significantly influence tissue development, differentiation, and cell viability [8]. Laminin is structurally composed of α, β, and γ trimers and has a remarkable capability to sustain human pluripotent stem cells (e.g., embryonic stem cells/iPS cells) in culture over extended durations [9,33,34,35]. Within the context of the NP, the laminin γ1 chain and α6 subunit are prominently expressed in the NP of juvenile pigs [36]. Previous studies have substantiated the role of laminin in orchestrating vital cellular processes such as survival and motility when present within the ECM of juvenile NP cells [36,37,38,39]. Laminin 511, comprising α5, β1, and γ1 chains [40], in particular, has been documented as a potent modulator, intricately involved in governing cell adhesion, migration, proliferation, differentiation, and overall cell survival [34]. However, the utility of laminin-based coating materials, such as Matrigel, is limited in the fields of cell therapy and regenerative medicine due to the unclear composition of these products and their derivation from xenogeneic sources. These factors create regulatory hurdles in the production and application of these products as potential advanced therapy medicinal products [41]. In response to these challenges, we highlight the emergence of iMatrix-511, a human recombinant laminin fragment meticulously purified to retain only the active site of laminin 511 [35]. This groundbreaking development has paved the way for monocultures where iMatrix-511 serves as the predominant basement membrane constituent, acting as a robust scaffold due to its exceptional cell adhesion properties [35]. Its application has been found in multiple fields, in particular for the production of iPS cell-derived products [42], such as nephron progenitor cells [43], myocytes [44], and keratinocytes [45], as well as for enhanced notochordal cell type retention [10]. This practical application to cell culture methods eliminates the need for 3D culture environments, repeated (expensive) growth factor addition, or the formation of complex regulatory hurdles. It poses a promising addition to culture procedures, enhancing cellular yields from scarce tissue sources. However, whether this relevant coating material could support the maintenance of Tie2-expressing NP progenitor cells remained unexplored. As such, in this study, we aimed to examine the potential of iMatrix-511 as a new cell-culture dish coating constituent to support the maintenance and expansion of human Tie2-expressing NP progenitor cells, as we hypothesized that the laminin-511 fragment could support the maintenance of NP progenitor cell phenotypes.

## 2. Results

### 2.1. Laminin-511 Coating Enhances NP Cell Proliferation Potency

**Figure 1:**
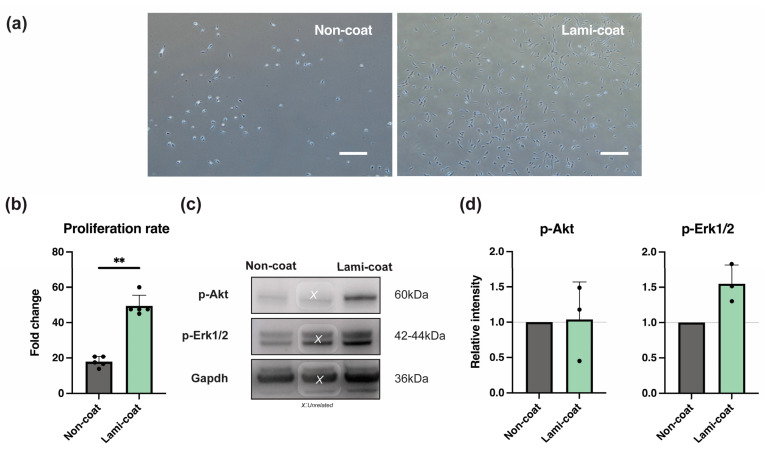
Assessment of cell morphology and proliferation (**a**) Morphological assessment of cultured nucleus pulposus (NP) cells for 6 days on either standard (Non-coat) or iMatrix (Lami-coat) coated dishes (Scale bar represents 1 µm). (**b**) Proliferation rates of cells cultured on both plate types (**c**) A Western blot example aimed to evaluate phosphorylated-Akt and phosphorylated-Erk1/2 levels. (**d**) Quantification of Western blot phosphorylated-Akt and phosphorylated-Erk1/2 levels for NP cells cultured on Non-coat and Lami-coat. Note: samples marked with X involve samples from an unrelated study simultaneously applied to the Western blot but have no relation to the presented study. ** indicates a *p* < 0.01 between the indicated groups. For a complete overview of Western blots, we refer to the Supplemental Files.

Human NP cells obtained from surgically extracted disc tissue were isolated using previous published methods [19,22] and were subsequently cultured on plates with iMatrix-511 coating (Lami-coat) or without coating (Non-coat). Figure 1a illustrates a distinct difference in cell morphology following culture, where Lami-coat dishes gave rise to spindle-shaped cells (specific NP cell characteristics [46,47]) and an overall larger cell size. Additionally, the microscopic images indicated a significant increase in cell numbers compared to the Non-coat condition. Furthermore, as a general observation, we noted that Lami-coat cells presented stronger adhesion to the dish during cell collection. Quantification of cell yields confirmed a significant increase in proliferation for cells cultured on iMatrix-511 (Non-coat 17.9 ± 2.9 fold change compared to 49.5 ± 6.0 in the Lami-coat, *p* < 0.001) (Figure 1b). Furthermore, analysis of extracellular signal-regulated kinase 1/2 (Erk1/2) and Ak strain transforming (Akt) phosphorylation (key signaling molecules involved in cell growth and survival [34,37,38]) through Western blotting showed a trend of enhanced activation of both Erk1/2 (*p* = 0.070) and Akt (*p* = 0.910) pathways. (Figure 1c,d).

### 2.2. Laminin-511 Coating Strongly Increases Tie2-Expressing NP Cell Yields

**Figure 2:**
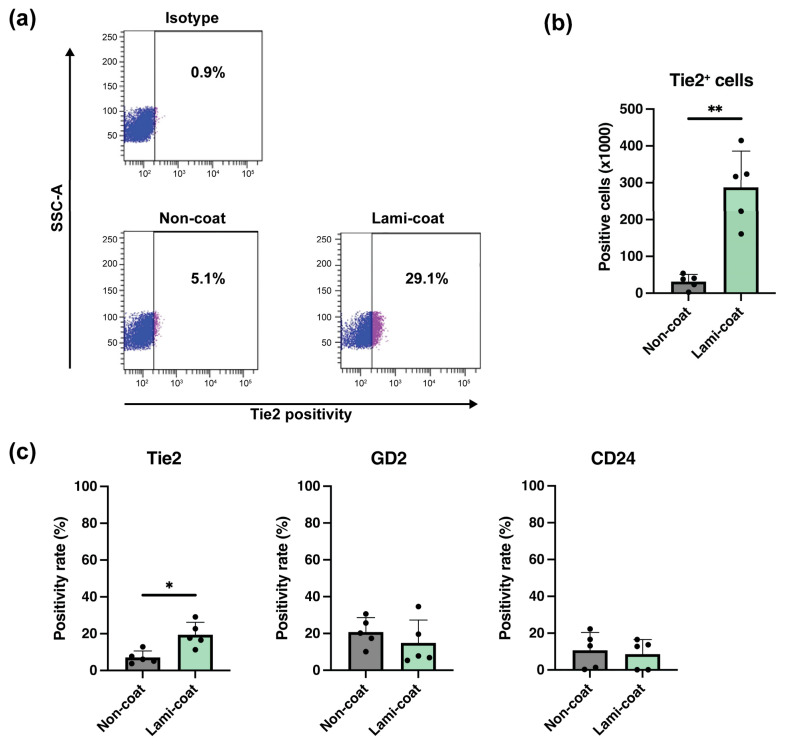
Flow cytometry analysis of cell surface markers to determine the maintenance of the nucleus pulposus progenitor cell phenotype (**a**) Example of flow cytometry gating for Tie2 positivity of cultured nucleus pulposus (NP) cells for 6 days on either standard (Non-coat) or iMatrix (Lami-coat) coated dishes, where gating plots show Tie2^-^ (blue) and Tie2^+^ (purple) measurements. (**b**) Total yield of NP cells positive for Tie2 after Non-coat or Lami-coat culture. (*n* = 5) (**c**) The positivity rate of Tie2, GD2, and CD24 of NP cells following Non-coat or Lami-coat culture. (*n* = 5) * and ** indicates a *p* < 0.05 and *p* < 0.01 between the indicated groups respectively.

Following the culture of NP cells, flow cytometry analysis was used to determine the retention of NP progenitor cells by determining the rate of Tie2, disialoganglioside (GD2), and CD24-expressing NP cells [19]. Lami-coat was able to significantly increase the absolute numbers of Tie2-expressing cells compared to Non-coat. Specifically, Non-coat conditions resulted in on average 36,260 (±12,255) Tie2^+^ cells compared to 287,745 (±98,125) Tie2^+^ cells after Lami-coat culture (*p* = 0.004), an almost 8-fold increase. (Figure 2a,b) In part, this increase can be explained by an increase in overall cell numbers by Lami-coat (Figure 1c), but Lami-coat also supported higher retention of Tie2-expressing cells, with an average positivity rate of 19.4% (±6.8%), compared to only 7.1% (±3.5%) in the Non-coat group (*p* = 0.033), a 2.7-fold increase. (Figure 2c) No evident differences in the expression of GD2 and CD24 (specific markers of differentiating NP cells [19]) were observed as a result of the applied culture conditions (Figure 2c).

### 2.3. Laminin-511 Supports Enhanced Type II Collagen Production

**Figure 3:**
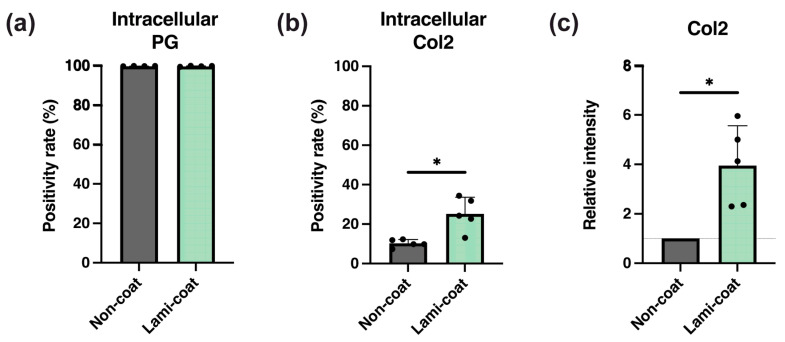
Assessment of extracellular matrix production potency of cultured nucleus pulposus (NP) cells for 6 days on either standard (Non-coat) or iMatrix (Lami-coat) coated dishes. Rate of cells presenting with positive intracellular staining against (**a**) proteoglycans (PG) and (**b**) type II collagen (Col2). (**c**) Western blot analysis analyzing levels of Col2 levels * indicated a *p* < 0.05 between the indicated groups (*n* = 5). Dotted line indicates relative intensity of control samples.

The expression of major ECM components Col2 and PG was assessed through flow cytometry and intracellular staining of precursor proteins to determine the potential of the obtained cells in NP-ECM production [22]. PG staining revealed no clear benefit for Lami-coat culture conditions, as both culture plates resulted in cell products predominantly positive for PG. (Figure 3a) On the other hand, Lami-coat was able to significantly increase the fraction of Col2-producing NP cells. Specifically, we found 10.2% (±1.8%) in the Non-coat condition and 26.2% (±7.5%) in the Lami-coat condition (*p* = 0.01, more than doubling the fraction of positive cells (Figure 3b), and higher than the previously reported range of <5–25% by Sako et al. [22]). Further assessment via Western blot confirmed enhanced Col2 production. Lami-coat cultures resulted in a significantly 3.9-times (±1.6) higher level than Non-coat cultures (Figure 3c, *p* < 0.015).

### 2.4. Colony Formation Rate Increased by Laminin-511

**Figure 4:**
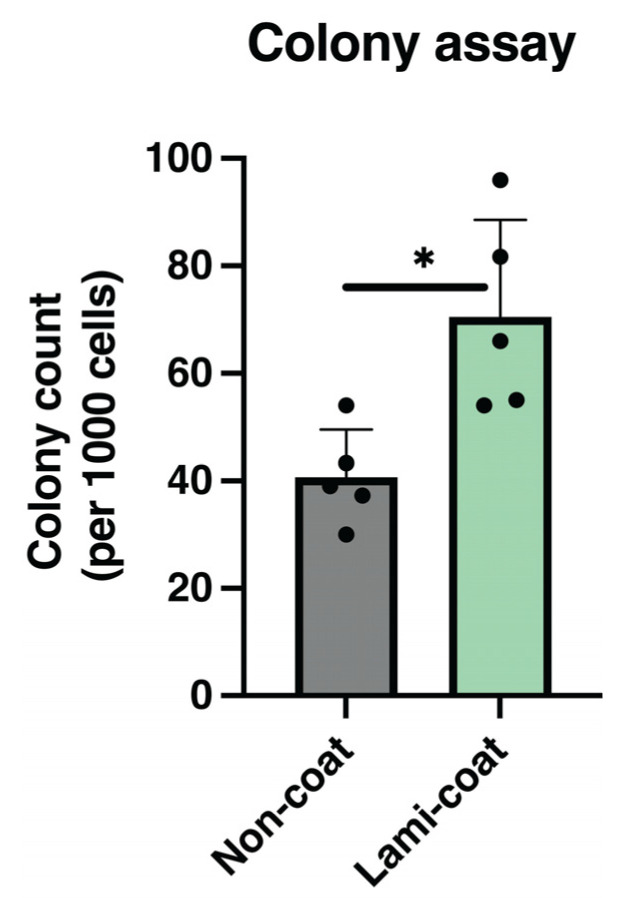
Quantification of the number of spherical colonies forming from nucleus pulposus (NP) cells cultured for 6 days on either standard (Non-coat) or iMatrix (Lami-coat) coated dishes, followed by a methylcellulose colony-forming assay. * Indicated a *p* < 0.05 between the indicated groups. (*n* = 5).

The self-renewal functionality of NP cells was evaluated using colony assays. Cells obtained following Non-coat and Lami-coat cultures were encapsulated in methylcellulose at 1000 cells mL*^−^*^1^ and subsequently cultured for 10 days [19]. Here, the Lami-coat was able to significantly increase the rate of established spheroid colonies. (Figure 4, *p* = 0.017) Specifically, Lami-coat cultured cells gave rise to an average of 70.53 (±18.08) colonies per 1000 cells, compared to only 40.73 (±8.84) colonies from the Non-coat condition, a 1.7-fold increase.

### 2.5. Integrins Are Crucial in Laminin-511-Mediated NP Progenitor Cell Retention

**Figure 5:**
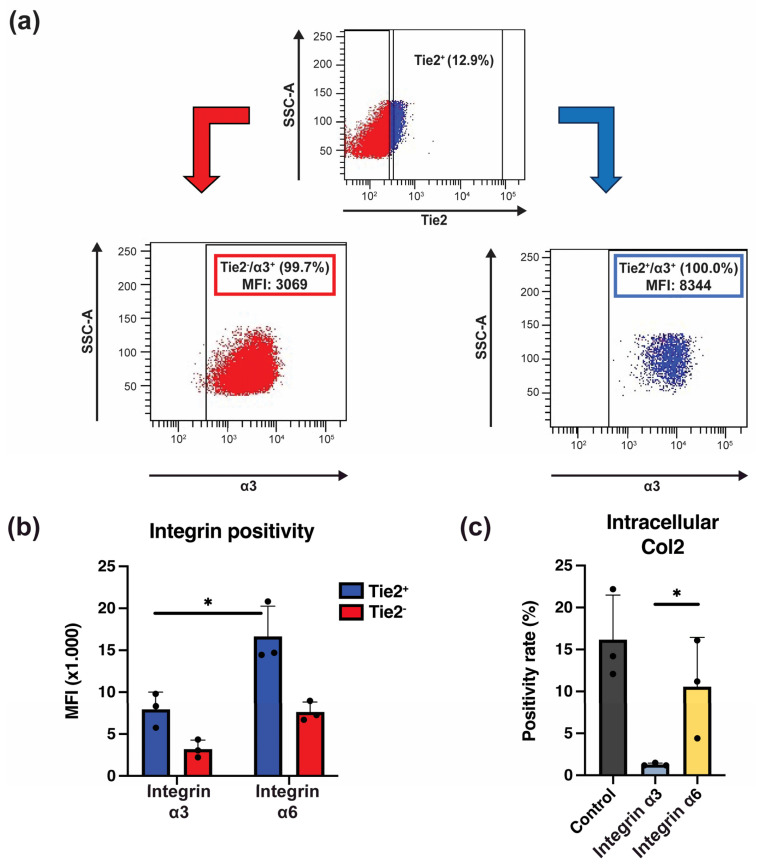
Assessment of integrin-α3 and -α6 involvement in the beneficial outcomes derived from laminin coating. (**a**) Example of a gating procedure in flow cytometry analysis involving sorting Tie2-positive (blue) and Tie2-negative (red) nucleus pulposus (NP) cells (**b**) Quantification of staining intensity against integrin-α3 and integrin-α6 in Tie2-positive and Tie2-negative NP cell populations. (**c**) Positivity rate of NP cells for intracellular Col2 following blocking of integrin-α3 or integrin-α6 compared to NP cells not subjected to any blocking antibody. * Indicated a *p* < 0.05 between the indicated groups. (*n* = 3).

Finally, we evaluated the expression and role of integrin receptors expressed by NP cells in relation to the beneficial effects observed by Lami-coat. Specifically, we focused on subunits α3 and α6, which are thought to be the key laminin receptors expressed by NP cells [36]. Our study confirmed their expression by NP cells. Moreover, comparing the differences in intensity of α3 and α6 expression between Tie2^+^ and Tie2^−^ NP cells revealed that Tie2^+^ NP progenitor cells presented a higher density of α3 and α6 compared to Tie2^−^ NP cells. (Figure 5a) Specifically, Tie2^+^ NP cells presented about a 2.5-fold higher intensity of α3 compared to Tie2^−^ NP cells. Similarly, intensity for α6 was 2.2-fold higher. Both cell populations had a higher α6 expression than α3 (Figure 5a), suggesting the importance of α6 for NP cell maintenance. To confirm this, we blocked each specific integrin subunit and assessed its impact on Col2 production potential. Blocking of either integrin α3 or α6 resulted in a decline of Col2 producing cells (Figure 5b), although surprisingly, α3 blocking resulted in a complete deterioration (1.3 ± 0.2%) of Col2 positive cells, while α6 blocking only showed a slight reduction (10.6 ± 5.9%) in the proportion of Col2 positive cells (Figure 5b).

## 3. Discussion

The purpose of this study was to analyze whether iMatrix-511 can be used to maintain the in vitro presence of Tie2-positive NP progenitor cells during the expansion culture process, which will be essential for the manufacturing of NP progenitor cell-based medicinal products. In this study, it was confirmed that culturing NP cells on iMatrix-511-coated culture dishes significantly enhanced their proliferation rate, which was associated with activation of the Erk1/2 and Akt signaling pathways. (Figure 1) The Erk1/2 pathway is a downstream signaling molecule of the mitogen-activated protein kinase signaling cascade, is also known to be involved in the production of inflammatory cytokines, and has been connected with regulating NP cells and ECM-binding [48]. On the other hand, the Akt pathway functions as a switch between cell death and cell survival [49,50]. It has been reported that activation of the Akt pathway in NP cells is involved in the enhancement of cell adhesion and inhibition of apoptosis [49,50]. Moreover, previous work has highlighted both pathways in relation to NP cell proliferation and Tie2 cell retention [22]. As iMatrix-511 was able to enhance Erk1/2 and Akt activation, it is reasonable to suggest that the observed enhanced proliferation is associated with an activation of these pathways through the binding of NP cell integrin subunits to the iMatrix-511 components (Figure 1).

Integrin subunits are expressed in vertebrates, and in total, 15 different laminin isoforms formed from α-chains (α1–α5), β-chains (β1–β3), and γ-chains (γ1–γ3) have been implicated in the regulation of cell survival and motility in the ECM of juvenile NP cells [34,51]. Moreover, the expression of laminin isoforms, which result from the assembly of these distinct chains, exhibits significant variation contingent upon the specific tissue type and developmental stage [34,51]. Chen et al. reported that NP cells express laminin α5 chain, laminin receptors (integrin α3, α6, β4 subunit, and CD239), and related binding protein CD151 at high levels, suggesting that the interaction of laminin with NP cells may be an important contributor to IVD biology [36]. Laminin-functionalized hydrogels have shown effectiveness in regulating NP cell phenotypes. For example, the work of Speer et al. [52] highlighted that the application of laminin-mimicking peptides in a poly(ethylene-glycol) hydrogel was able to promote NP cell phenotype retention. Thereby, emphasizing the general potential of laminins to direct NP cell behavior. In the present study, culture on the iMatrix-511 coating was able to significantly enhance Tie2 expression, suggesting that laminin-511 is significantly involved in the maintenance and survival of NP cells. Tie2-expressing NP cells have an intrinsically high proliferative potential and differentiation capacity; however, previous work has highlighted their deterioration with aging and catabolic conditions [18,19,22,27,53]. In addition, cells tend to lose their progenitor traits after repeated passaging, especially NP cells [19,27,29,46,54], making it difficult to maintain and create high numbers of potent (progenitor) cells for potential therapeutic products. As primarily Tie2^+^ NP progenitor cells can only be extracted at impactful rates from relatively young donors and access to such tissues is scarce, ensuring successful retention and, ideally, expansion of these cells is critical for their development as potential (allogenic) cell transplantation products. As such, by applying iMatrix-511, we could obtain a significant 7.9-fold increase in the number of Tie2^+^ NP progenitor cells as well as general NP cell yields. Meaning that our culture method is able to produce a larger number of general NP cells, of which more NP cells are Tie2-positive, which in turn produced an almost 8-fold increase in Tie2-expressing NP cell numbers for potential medicinal or research purposes. This increase is higher than previous attempts, e.g., alginate beads application (1.8-fold increase [24]), spheroid-based suspension cultures (3.6-fold increase [20]), or application of whole tissue culture with fibroblast growth factor 2 (FGF2) stimulation (7.5-fold increase [22]). Emphasizing the potency of laminin-511 coating strategies to facilitate the expansion of these valuable NP progenitor cells. Here, the iMatrix-511 also has the benefit of ease of use as it does not require culturing cells in a 3D environment and specialized methods for cell extraction from alginate beads or ECM environments, potentially reducing production costs. Moreover, not only did our method show retention of Tie2^+^ cells, but these cells were similarly able to maintain their regenerative potential, as indicated by their active production of ECM components. In our study, we observed that regardless of the culture conditions, over 90% of harvested cells were actively involved in producing PG. Opposingly, Col2 positivity and production were significantly increased by cells subjected to the Lami-coat condition, further promoting the concept that iMatrix-511 was able to enhance NP progenitor cell features in culture.

In this study, we examined the binding of NP cells to laminin 511, and we wanted to determine which integrin isoforms were responsible (in part) for the beneficial effects seen by this cell-matrix connection. iMatrix-511 is a recombinant protein with an identical sequence to laminin 511, and for NP cells, it is related to its primary receptor, α6β1 integrin [55]. We assessed the impact of α3 and α6 by blocking their functionality and assessing the impact on Col2 production. Surprisingly, despite much higher expression of integrin α6 on NP cells, the main detrimental effects were seen following the blocking of subunit α3. Although previous studies have identified α6β4, α3β1, and α7β1, including α6β1, as major laminin receptors in various cell types, our study suggests that α3β1 may play a pivotal role as a laminin receptor in NP cells, particularly in relation to laminin-511 [56]. Moreover, considering the large range of laminin and integrin types present in NP cells, it is expected that other unexplored integrin subtypes are involved in Tie2-posivity and general NP cell phenotype maintenance. The observation of almost complete loss of Col2-producing cells following α3-blocking does underline the importance of α3. Alternatively, integrin α3 might be an interesting marker for Col2-producing NP cells. Further investigations into the interaction between laminin 511 and its receptors in NP cells are underway and will shed light on the mechanisms underlying NP cell-induced degenerative changes in the IVD. It is worth noting that the examination of integrin binding and the regulatory mechanisms between human NP cells and iMatrix-511 represent intriguing topics for future research.

Finally, our results and previous studies have suggested that the evaluation of intracellular Col2 expression levels in conjunction with Tie2^+^ NP progenitor cells and spheroid colony-forming ability may be more effective markers to evaluate highly functional NP cells [22]. Previous studies examining the application of spheroid colony-forming NP cells as cellular therapeutics have highlighted their potential in pre-clinical animal models as effective and safe strategies to limit disc degeneration [57]. These cells are currently undergoing assessment for the treatment of discogenic LBP in in-human trials [58,59] and interim results are announced as promising [60]. However, to further advance the application of specifically highly potent NP progenitor cells remains a challenge, and critical production design is needed to enable successful clinical and market translation, including aspects of production scalability, quality consistency, regulatory approval, intellectual property protection, and product safety/purity assurances [61]. Our study demonstrates that culturing with iMatrix-511 purifies highly active NP cells that maintain their ECM-production potency and thereby could potentially enable the production of large numbers of cells that maintain their regenerative functionality. The results of this study suggest that purification of a large amount of NP cells from a small amount of relatively young and healthy IVD tissue is possible, thereby expanding the opportunities for NP progenitor cells to be used as regenerative medicinal agents.

One limitation of our study is that we did not assess the baseline values, such as the initial Tie2 positivity of collected NP cells. This omission was due to practical constraints, including the scarcity of tissue sources and limited cellular yields. Therefore, we were unable to determine whether the enhanced Tie2^+^ population involved an increase in retention or enhanced production of the Tie2^+^ populations. Additionally, our study focused on the production of general PG and Col2, but we did not examine specific PG types or other NP-ECM constituents, such as elastin. Therefore, further research is needed to determine the effects of laminin-511 on these matrix components. Furthermore, our study is a preliminary investigation, and future work is required to determine how the NP cells derived from Lami-coat culture function as a regenerative transplantation product in relevant disc degeneration models.

## 4. Materials and Methods

The study obtained approval from the Institutional Review Board for Clinical Research at Tokai University with application number 17R-173. This signifies that all research procedures outlined in the study adhered to the ethical and safety guidelines set forth by our institution. Human IVD tissues were obtained from patients undergoing lumbar disc herniation surgery upon obtaining their informed consent (*n* = 13, mean age: 18.8 ± 2.3; Table 1).

### 4.1. Methods of NP Cell Isolation

NP cells were isolated as previously described [19] and methods are described in accordance with recommendations stated by the ORS Spine community [46]. Collected IVD tissues were washed with saline, and a careful selection of gelatinous NP tissue was separated and processed further. The isolated NP tissue was shredded into small pieces and then centrifuged (966 g, 5 min, 4 °C) with fetal bovine serum (FBS, SAFC, 12003C-500ML, 19B239) and minimum essential medium α (MEMα, FUJIFILM, MEMα with L-Glutamine and Phenol Red, 135-15175). After centrifugation, the tissue was resuspended in TrypLE Express (Thermo Fisher Scientific, Tokyo, Japan, TrypLE™ Express Enzyme (1×), phenol red, 12605093) (37 °C, 5% O_2_, 21% CO_2_). The suspension was incubated with slow swirling (37 °C, 30 min). The tissue was then checked for lysis and centrifuged (340 G, 5 min, 4 °C). The tissue was then resuspended in 10% FBS-MEMα and 0.25 mg/mL collagenase P (Roche Diagnostics GmbH, Tokyo Japan, Collagenase P, 11213857001) and incubated (37 °C, 2 h). After treatment, the tissue suspension was centrifuged once more (340 G, 5 min, 4 °C), resuspended in 10% FBS-MEMα, and transferred onto a 40 μm cell strainer (FALCON A Corning Brand, New York, NY, USA, Cell Strainer, 40 μm Nylon, REF: 352340). Cells were cultured for approximately 1 week on regular culture plates for one passage at a density of 3 × 10⁴ cell/cm^2^ in culture media (10% (FBS/MEMα/DMEM media) as previously described [22]) at 37 °C, 5% O_2_, and 21% CO_2_. Cells were harvested by washing them with PBS once and applying TrypLE Express at 37 °C, 5% O_2_, and 21% CO_2_ for 5 min. Collected cells were obtained in 10% (*v*/*v*) FBS MEMα, counted, and directly applied for experimentation.

### 4.2. Laminin-Coating

55 µL of 0.5 µg/mL iMatrix-511 (Nippi, Tokyo, Japan, iMatrix-511, Version 010) solution was added to a 100 mm dish (IWAKI 3020-100, IWAKI, Sayama, Japan, 100 mm Tissue Culture Dish) and incubated in a humidified chamber (37 °C, 5% O_2_, 21% CO_2_, 1 h). As a control, identical plates were similarly treated with equal volumes of PBS. After incubation, the coating reagents were removed, and culture medium was added without washing the dish. Cells were seeded directly onto the plates at a density of 3 × 10⁴ cell/cm^2^ and kept at 37 °C, 5% O_2_, and 21% CO_2_ without passaging.

### 4.3. Cell Viability and Counting

After day 6 of culture, the cells were collected using TrypLE as described above. Next, using Trypan blue (Fujifilm Wako Pure Chemicals Corporation, Osaka, Japan, 0.4% *w*/*v*) and a blood cell counting plate, overall cell numbers and viability rates were assessed under a phase contrast microscope (Nikon Corporation, Tokyo, Japan, Model ECLIPSE Ti2-U, 543097).

### 4.4. Western Blot Analysis

The expression level of Col2 at day 5 of culture was detected through Western blot analysis. The supernatant was collected from each culture dish and washed using Tris Buffer Saline (1M Tris/HCl: 250 mL, NaCl: 87.66 g, Tween-20: 50 mL, MilliQ: 9700 mL). After washing, cells were collected using TrypLE Express, MilliQ, and RIPA Buffer (25 mM Tris-HCL, 150 mM NaCl, 1% NP-40, 1% deoxycholic acid, and 0.1% sodium dodecyl sulfate), 200 mM PMSF (Cell Signaling TECHNOLOGY 34.84 mg #8553) in lysis buffer, and incubated on ice for 1 h. The cells were then centrifuged (12,000 rpm for 5 min at 4 °C), and the proteins extracted from the supernatant were collected. Cells were incubated in a microplate reader (MOLECULAR DEVICES, VersaMax™) using DC protein assay reagent (BIO-RAD, DC Protein Assay Reagent #1310-73-2, #5000114, #500-0115), Absorbance Microplate Reader (100–240 V 4 A 50–60 Hz, 250 V, T4.0 AH) quantified by (700 nm).

The extracted samples were crushed (5 cycles, 30 sec, high power) using an ultrasonic crushing device (BIORUPTOR, Sonic Bio Inc., Samukawa, Japan). Extracted samples were mixed with 4 × Buffer and 2 ME (Melcapto ethanol) and heated (100 °C, 5 min) to denature SDS. Polyacrylamide gel (Invitrogen NuPAGE 4–12% Bis-Tris Gel BoltTM4–12% Bis-Tris Plus 1.0 mm × 12 wells) and MES Running Buffer (Invitrogen NuPAGETM, MES SDS Running Buffer [20×] NP0002) was used to separate protein lysates (200 V, 30 min). After electrophoresis, the proteins were transferred on an electrophoresis system (20 V, 60 min) using a PVDF membrane (Merck Millipore Ltd., Burlington, VT, USA, Immobilion^®^-P, Transfer Membrane Filter Type; PVDF, Pore Size; 0.45 μm). Membranes were washed (10 min, 3 times), blocked with 3% bovine serum albumin (Merck Millipore Ltd., Probumin^®^ Bovine Serum Albumin Universal Grade) (37 °C, 30 min), and then washed with Buffer G (0.1% gelatin, 0.1% BSA, 1 mM MgCl2, 0.1% NaN3 in PBS), then diluted with primary antibodies Phospho-Akt Ser473 Antibody (Cell Signaling Technology 1:1000, Danvers, MA, USA), Phospho-44/42 Erk1/2 (Cell Signaling Technology 1:1000), Mouse anti-Collagen II 5825 (NOVUS 1:1000), monoclonal antibody GAPDH clone (Rabbit anti-GAPDH Polyclonal antibody (Sigma-Aldrich 1:1000, Burlington, VT, USA) and reacted overnight (4 °C) with secondary antibody (ECL^TM^ Anti-mouse IgG, Horseradish Peroxidase-linked F(ab’2) 1:2000) diluted in TBS-T and incubated (room temperature, 1 h) with detection reagent (Millipore Immobilon^®^ Western Chemiluminescent HRP Substrate 500 mL, Merck Millipore Ltd., Burlington, VT, USA) to detect immunoreactive bands. Protein signals were quantified by a multi-imaging system (Vilber Bio Imaging FUSION Solos (manual focus model Evolution Capt) Paris, France). For all analysis, GAPDH was used as a loading control and for internal normalization.

### 4.5. Flow Cytometry Analysis

NP cells were analyzed using a FACS-Calibur flow cytometer (BD Biosciences (Franklin Lakes, NJ, USA), BD FACS-Canto^TM^ II Flow Cytometer, 338962) following previous work [19]. Cells were treated with primary antibodies IgG1 mouse -FITC (Beckman Coulter, Brea, CA, USA, IM2475), IgG1 mouse -APC (BD Biosciences, A07796), IgG1 mouse -PE (BD Biosciences, IM2475), anti-hTie-2 APC Conjugated Mouse IgG1 (R&D SYSTEMS, Minneapolis, MN, USA, FAB3131A), PE Mouse Anti-Human Disialoganglioside GD2 (BD Biosciences, 562100), FITC Mouse Anti-Human CD24 (BD Biosciences, 555427) (10 μg/mL each) and incubated on ice (1 h). Double-stained cells were incubated with primary antibodies IgG1 mouse -APC (BD Biosciences, A07796), IgG1 mouse -PE (BD Biosciences, IM2475) (control, 10 μg/mL each), anti-hTie-2 APC Conjugated Mouse IgG1 (R&D SYSTEMS, FAB3131A), PE Anti-human CD49c (Integrin α3; BD Pharmingen TM, 556025) and PE Anti-human CD49f (Integrin α6; BD Pharmingen TM, 555736) (10 μg/mL each), each were added simultaneously and kept on ice (1 h). Cells were then washed with PBS and subjected to the assay. Only viable cells were targeted during the assessment, using a viable gate (propidium iodide-negative). Cell sorting methods and monoclonal antibodies used for analysis were performed as previously described [19].

For intracellular PG and Col2 stainings, harvested NP cells were fixed and permeabilized using IntraPrep Permeabilization Reagent (Beckman Coulter, A07803). IntraPrep 1 (100 μL) was added to the NP cells and incubated in a water bath (37 °C, 10 min). After centrifugation (340 g, 5 min, 4 °C), the supernatant was collected, and 100 μL of IntraPrep 2 was added and incubated (room temperature, 1 h). After incubation, samples were frozen (−80 °C) and thawed in a water bath, and this was repeated three times. The next samples were treated with NOVUS Mouse anti-Collagen II 5825 or Sigma-Aldrich Anti-Cartilage Proteoglycan Antibody, adult, clone EFG-4 MAB2015, and kept overnight (4 °C). The next day, the samples were washed with PBS and incubated (room temperature, 1 h) with a secondary antibody (Becton, Dickinson, and Company, Franklin Lakes, NJ, USA, Goat Anti-Mouse Ig, FITC, REF: 349031) before flow cytometry measurement.

### 4.6. Colony Formation Assay Method

Following the recommendations set in Sakai et al. [19], a total of 1000 Lami-coat and Non-coat-derived cells (*n* = 5) were cultured in 1 mL of the methylcellulose hydrogel product Methocult (ST-04230 MethoCult H4230, STEMCELL Technologies (Vancouver, BC, Canada)) for 10 days at 37 °C with 5% O_2_ and 21% CO_2_. Methylcellulose is a hydrogel product often used to determine anchor-independent colony formation, proliferation, and general stemness of stem cells or cancer cells [33,62,63]. Next, the number of spheroid colonies formed per ml of MethoCult was manually counted using an inverted phase contrast microscope (Nikon Corporation, Model ECLIPSE Ti2-U).

### 4.7. Integrin Blocking Assay

Cells were obtained following Non-coat conditions according to the NP cell collection method described above and were subsequently incubated with 30 μg/mL primary antibody mouse IgG1 -PE (Isotype control; BD Biosciences, IM2475), 30 μg/mL PE Anti-human CD49c (Integrin α3; BD Bioscience Pharmingen^TM^, 556025), or 30 μg/mL PE Anti-human CD49f (Integrin α6; BD Bioscience Pharmingen^TM^, 555736) and kept on ice (1 h). Cells were washed with PBS and assayed in 0.5% BSA + α (0.5% BSA (Bovine Serum Albumin Solution, Sigma-Aldrich, 9048-46-8), MEMα) overnight. Next, samples were incubated with secondary antibodies (Goat anti-Mouse IgG Recombinant Secondary Antibody, Alexa Fluor 488 (Invitrogen A28175)) to determine intracellular Col2 as previously described.

### 4.8. Statistics and Visualization

Statistics in this study were performed using GraphPad Prism v9.0.1(128) (GraphPad Software Inc., Boston, MA, USA). Data set normality was confirmed via the Shapiro–Wilk test. For data involving two groups, a paired *t*-test was employed. For comparisons of more than two groups, a two-way ANOVA was used for statistical analysis. A *p*-value below 0.05 was considered statistically significant. All data are presented as a mean value, with variability indicated as a standard deviation. Figures are designed using GraphPad Prism v10.0.2 (GraphPad Software Inc.), and figures are created by Adobe Illustrator version 27.8.1 (Adobe Inc., San Jose, CA, USA).

## 5. Conclusions

In summary, we have shown that it is possible to successfully perform and enhance cell expansion culture of human NP cells while maintaining progenitor cell traits through the application of clinically relevant laminin-511-based iMatrix coating. In the future, we aim to evaluate the relationship between specific laminin subunits and NP cell phenotype maintenance and further explore the potential of laminin-mediated instructions to promote NP cell and NP tissue regenerative strategies. Specifically, we aim to identify regulatory targets to promote the regenerative potential of NP (progenitor) cells or laminin-based products for cellular therapeutics.

## Figures and Tables

**Table 1:** Overview of clinical tissue samples obtained and applied in this study.

Patient	Sex	Age (y)	Pathology	Experimental Use
A22	M	22	LDH	FCM, CFA, WB
T20	M	20	LDH	FCM, CFA
A18	M	18	LDH	FCM, CFA
T18	M	18	LDH	FCM
T17	F	17	LDH	FCM
T16	M	16	LDH	FCM, CFA
A23	M	23	LDH	WB, CFA
T21	M	21	LDH	WB
A20	M	20	LDH	WB
A16	M	16	LDH	WB
K18	M	18	LDH	WB
A16	M	16	LDH	WB
T19	M	19	LDH	FCM

Abbreviations: CFA: colony-forming assay, FCM: flow cytometry, LDH: lumbar disc herniation, and WB: Western blot.

## Data Availability

All data is pesented in the manuscript. Additional data can be requested from the corresponding authors upon reasonable request.

## Supplementary Materials

The following supporting information can be downloaded at: https://www.mdpi.com/article/10.3390/ijms242316713/s1.

## Glossary

Akt: Ak strain transforming, Col2: Type II collagen, ECM: Extracellular matrix, Erk1/2: Extracellular signal-regulated kinase 1/2, FBS: Fetal bovine serum, GD2: Disialoganglioside, Lami-coat: iMatrix-511 coating, LBP: Low back pain, iPS cell: Induced pluripotent stem cell(s), IVD: Intervertebral disc(s), MEMα: Minimum essential medium-α, NP: Nucleus pulposus, Non-coat: Non-coated dishes, PG: Proteoglycan(s), and Tie2: Angiopoietin-1 receptor tyrosine kinase.
