# Supplementary material for: Recombinant Laminin-511 Fragment (iMatrix-511) Coating Supports Maintenance of Human Nucleus Pulposus Progenitor Cells In Vitro
Source: Int J Mol Sci. 2023 Nov 24;24(23):16713. doi: 10.3390/ijms242316713 (PMC10706138; doi:10.3390/ijms242316713)
Supplement: Supplementary file 1 [file ijms-24-16713-s001.zip › ijms-2687577-supplementary.pdf]

Western blots for pErk1/2 and GAPDH (loading controls) for each of the three analyzed donors (A20, T21, A17; see table 1)

“Unrelated” refers to samples that were applied on the Western blot for a different and unrelated study.

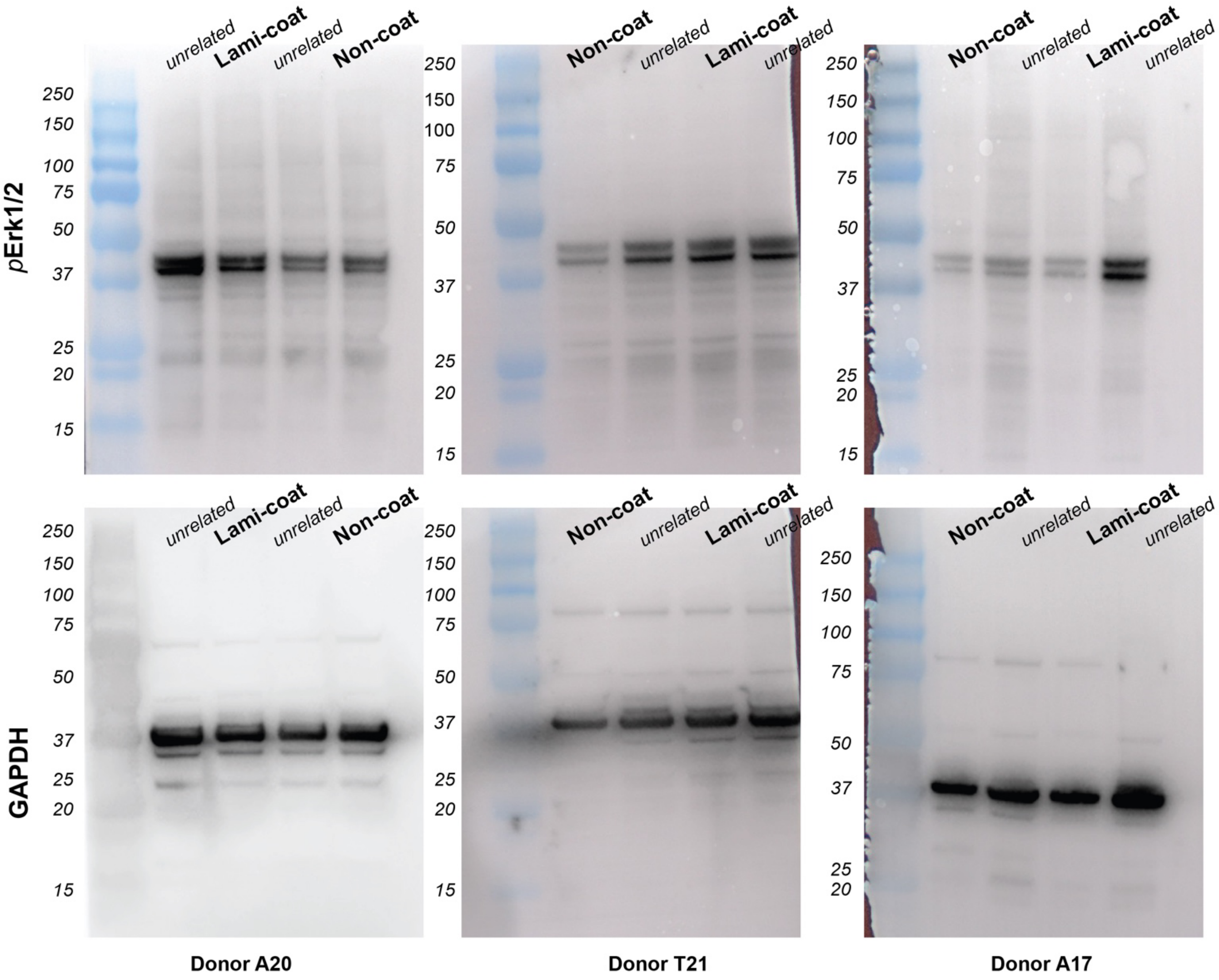

This supplemental file is in reference to:
Recombinant Laminin-511 Fragment (iMatrix-511) Coating Supports Maintenance of Human Nucleus Pulposus Progenitor Cells in Vitro. H Soma, D Sakai, Y Nakamura, S Tamagawa, T Warita, J Schol, E Matsushita, M Naiki, M Sato, and M Watanabe. (2023)

Western blots for pAkt and GAPDH loading controls for each of the three analyzed donors (A20, T21, A17; see table 1)

"Unrelated" refers to samples that were applied on the Western blot for a different and unrelated study.

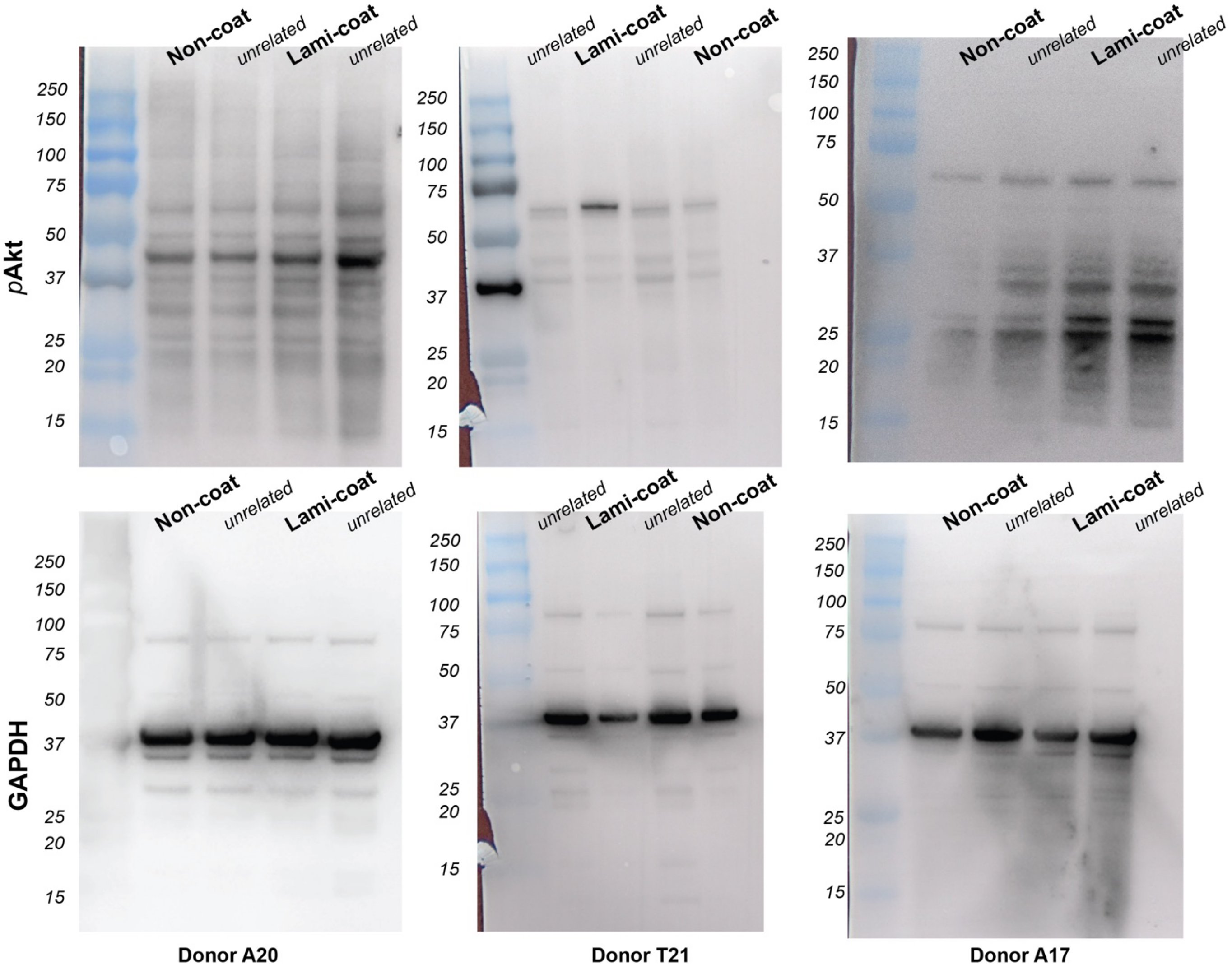

This supplemental file is in reference to:
Recombinant Laminin-511 Fragment (iMatrix-511) Coating Supports Maintenance of Human Nucleus Pulposus Progenitor Cells in Vitro. H Soma, D Sakai, Y Nakamura, S Tamagawa, T Warita, J Schol, E Matsushita, M Naiki, M Sato, and M Watanabe. (2023)
